# Falls risk perception measures in hospital: a COSMIN systematic review

**DOI:** 10.1186/s41687-023-00603-w

**Published:** 2023-06-26

**Authors:** Elissa Dabkowski, Karen Missen, Jhodie Duncan, Simon Cooper

**Affiliations:** 1 Institute of Health and Wellbeing, Federation University Australia, Northways Road, Churchill, VIC 3842 Australia; 2 Research Unit, Latrobe Regional Hospital, Traralgon West, VIC Australia; 3 Health Innovation and Transformation Centre, Federation University Australia, Berwick, VIC Australia

**Keywords:** Falls, Patient, Perception, Hospital, Fall prevention, Falls risk, COSMIN

## Abstract

Falls prevention in hospital continues to be a research priority because of the poor health outcomes and financial burdens that can arise. Recently updated World Guidelines for Falls Prevention and Management strongly recommend evaluating patients’ concerns about falling as part of a multifactorial assessment. The aim of this systematic review was to evaluate the quality of falls risk perception measures for adults in a hospital setting. This review was conducted using the Consensus-based Standards for the selection of health Measurement Instruments guidelines and provides a comprehensive summary of these instruments, including psychometric properties, feasibility and clinical recommendations for their use. The review followed a prospectively registered protocol, in which a total of ten databases were searched between the years 2002 and 2022. Studies were included if the instruments measured falls risk perception and/or other psychological falls constructs, if they were conducted in a hospital setting and if the target population contained hospital inpatients. A total of 18 studies met the inclusion criteria, encompassing 20 falls risk perception measures. These falls risk perception instruments were grouped into five falls-related constructs: Balance Confidence, Falls Efficacy/Concern, Fear of Falling, Self-Awareness and Behaviour/Intention. Two of the patient reported outcome measures (PROMs) received Class A recommendations (Falls Risk Perception Questionnaire and the Spinal Cord Injury-Falls Concern Scale); however, this rating is only applicable for the populations/context described in the studies. Thirteen PROMs received Class B recommendations, solidifying the need for further validation studies of these PROMs.

**Supplementary Information:**

The online version contains supplementary material available at 10.1186/s41687-023-00603-w.

## Introduction

Fall-related events are a major global public health issue resulting in approximately 684,000 deaths each year, with a further 172 million people impacted by a short or long-term disability due to a fall [2]. In a hospital environment, patient falls are one of the greatest sources of patient harm, with an estimated 700,000 to 1 million people falling each year in the United States of America alone [3]. The increasing economic burden to healthcare organisations from patient falls has been well documented and is expected to rise due to an ageing population [4]. There is no clear, single efficacious intervention for falls prevention in hospital, however partnering with patients and/or their families to develop individualised fall prevention plans is strongly recommended [5]. Collaborative decision-making between the patient and clinician results in greater patient satisfaction and improved health and safety outcomes [6]. Therefore, understanding the patients’ perspective creates an opportunity for health professionals to explore these influences, creating drivers for change [7].

Patient reported outcome measures (PROMs) can be used by health professionals to determine patients’ views of their symptoms, functionality and their health-related quality of life [8]. The use of PROMs enhances patient-clinician interaction, as patients are considered ‘the expert’ of the impact of interventions on their symptoms, quality of life and functional capacity [9]. PROMs in the form of fall risk perception measures may provide health professionals with the opportunity to capture patients’ perception in a clinical setting and to collaboratively develop suitable fall prevention plans. These instruments differ to that of physiological fall risk assessment tools (FRATs), which provide a rating or a score that reflects the patients’ propensity for falling. There is limited evidence on the predictive validity of falls risk screening tools for inpatients, especially those that are considered elderly [10]. In fact, high-quality evidence suggests that the use of scored FRATs do not lead to a reduction of fall rates in hospitals [11–13]. Updated world guidelines for falls prevention and management strongly recommend including an evaluation of patients’ concerns about falling, as part of a multifactorial falls risk assessment [14].

Fall risk perception measures have been developed over the years to measure various falls-related constructs. Examples of these include the falls efficacy scale (FES) [15], fear of falling questionnaire (FFQ) [16], activities-specific balance confidence (ABC) scale [17], the spinal cord injury-falls concern scale (SCI-FCS) [18], self-awareness of falls measure (SAFRM) [19] and more recently the self-awareness of falls in elderly (SAFE) scale [20] and the falls risk perception questionnaire (FRPQ) [21]. A previous scoping review identified the need for further investigation into these validated tools, as some studies have used these measures in a manner to which they were not intended [22]. Previous reviews have investigated fall-related psychological outcome measures [23, 24] and falls efficacy instruments for community-dwelling adults [25]. However, these reviews were not specific to an inpatient setting and a number of falls risk perception instruments have since been published. The Consensus-based Standards for the selection of health Measurement Instruments (COSMIN) offers a framework to systematically appraise and select instruments for use in clinical practice [26]. Evaluating and summarising the measurement properties reported for these individual measures provides an important contribution to the evidence-based selection of PROMs [27]. Therefore, the purpose of this review is to investigate and appraise inpatient fall risk perception measures using the COSMIN guidelines. The recommendations from this review will help to inform tool selection for falls prevention and management in hospitals.

## Aim

The overarching aim of this systematic review is to provide a comprehensive summary of the psychometric properties of fall risk perception measures for adults in a hospital setting. This review seeks to (1) evaluate the quality of falls risk perception instruments for use in adults; (2) provide recommendations for the feasibility of these measures in the context of fall prevention and management and- (3) identify any research gaps that would benefit from further inquiry. These aims were devised using the four key elements guided by Prinsen et al. [28], which includes the construct, the population, the type of instrument(s) and the measurement properties of interest.

## Method

### Design

This review follows the COSMIN guidelines, which provide a framework for evaluating measurement instruments and assessing the risk of bias of PROMs [26]. A protocol was registered with PROSPERO International prospective register of systematic reviews (registration no: CRD42022309582). This review also follows the preferred reporting items for systematic reviews and meta-analyses (PRISMA) checklist [1], given that the PRISMA-COSMIN guidelines are under development [27].

### Search strategy

The authors consulted a research librarian about the search strategy on three occasions in January and February 2022. A formal literature search was conducted by author ED in February and March 2022 of the following databases: Academic Search Complete, CINAHL Complete, MEDLINE, APA PsycINFO, APA Psyc Articles, Web of Science, SCOPUS, Cochrane library, PubMed and the search engine Google Scholar. The final search was conducted 12^th^ March 2022. The search was limited to peer-reviewed, full-text studies published in the English language between 2002 and 2022. A COSMIN review is usually conducted without a date restriction, however the authors opted to use a twenty-year time frame to establish the latest evidence, given the extensive nature of falls research. The databases were searched using a Boolean search strategy, which included key concepts and their variations and truncated symbols (see Additional file 1). All identified papers were analysed by their title, abstract, keywords and MeSH terms. The reference lists of identified papers were also searched to uncover additional studies. These search results were uploaded to Covidence database [29], a software program for screening systematic reviews for a blinded review of the studies. After de-duplication, authors ED and SC independently completed a title and abstract screen of all uploaded citations. In the event of uncertainty, author KM moderated the process until consensus was reached. The approved screened records were obtained in full text by author ED and further evaluated by the research team to determine their relevance to the review aims.

### Study selection

All study designs were eligible for inclusion if they related to instruments measuring fall risk perception and/or other various psychological fall constructs such as efficacy, awareness or fear of falling. COSMIN guidelines recommend to include all PROMs measuring one or more constructs of interest, rather than the most frequently used PROMs [30]. Therefore, given the broad definition of fall risk perception and associated psychological constructs, the authors discussed the suitability of the instruments before determining their eligibility for the review. The inclusion and exclusion criteria were developed by all four authors and consisted of pre-determined criteria. Articles that focused on physiological falls risk assessment tools were excluded, along with studies which did not include hospital inpatients as the target population. Therefore, studies conducted in residential care facilities, community-dwelling and outpatient settings were ineligible. For the studies that included mixed populations (both inpatient and outpatient adults), consideration was given if a subgroup analysis of both datasets was completed. Studies were included if they focused on a PROM development or adaptation of a falls risk perception measure. Studies were also included if they reported on the psychometric evaluation of measurement properties of a PROM, such as the structural validity or reliability. Cross-cultural adaptation and translational studies of falls risk perception measures were also eligible for inclusion if they were conducted in an inpatient setting. Letters, discussion papers and theses were also excluded.

### Data extraction and quality appraisal

Data extraction from the included studies was conducted and evaluated in accordance with the COSMIN Risk of Bias Checklist [31]. The purpose of conducting a quality appraisal in a systematic review is to assess the risk of bias or ‘trustworthiness’ of the included studies [31]. Data was extracted into prepared tables by author ED and co-verified by authors SC and KM for accuracy. All authors have experience in quality appraisal and instrument development, with author SC providing expert guidance of the appraisal.

Initially, a data summary table was developed, which detailed the author, year and country of study, the year of tool development and author (if applicable), the primary fall perception measure (otherwise known as the PROM) and construct, target population and cognitive status, setting, number of scale items, description of scale, interpretation of scoring, test completion time and recall period. Secondly, the content validity of each PROM was assessed using ten predefined COSMIN standards to determine the relevance, comprehensiveness and comprehensibility of the PROM for the context, population and construct [30]. This was completed through evaluating the quality of the original PROM development and any additional studies available on the PROM in this review. Each of the ten standards were rated as either ‘very good’, ‘adequate’, ‘doubtful’ or ‘inadequate’. Using the ‘worst score counts method’, the results of all available studies were qualitatively summarised to determine whether the overall content validity for each PROM is sufficient (+), indeterminate (?) or insufficient (−) [30].

The remaining measurement properties assessed from each PROM were structural validity (degree to which scores of a PROM are an adequate reflection of the falls construct), internal consistency (degree of interrelatedness among PROM items), cross-cultural validity (degree to which performance of items on a translated or culturally adapted PROM are an adequate reflection of the items of the original version)/measurement invariance, reliability (extent to which scores for patients who have not changed are the same for repeated measurements), measurement error (error of an individual’s score which is not attributed to true changes in the construct being measured), hypotheses testing for construct validity (consistency with hypotheses, outcome or aims stated in study) and responsiveness (ability of a PROM to detect change over time) [31, 32]. Additional file 2 details the measurement properties and definitions of these terms. Criterion validity was not assessed due to the varied nature of falls constructs and the current lack of gold standard for falls perception instruments. Similar to content validity, each of the measurement properties of the PROMs were assessed based on a risk of bias checklist and received ratings of ‘very good’, ‘adequate’, ‘doubtful’ or ‘inadequate’ [26]. An overall result for each measurement property was obtained by combining the results of all available studies in the review and rated as sufficient (+), indeterminate (?) or insufficient (−).

Finally, the results of all measurement properties from the PROMs were pooled and assessed using the Modified Grading of Recommendations Assessment, Development and Evaluation (GRADE) approach. The modified GRADE approach is determined by (1) risk of bias; (2) inconsistency; (3) imprecision and (4) indirectness, where the overall quality of evidence was rated as high, moderate, low or very low [26]. The quality of evidence indicates the trustworthiness of the results, as assessed by the authors. Grading of the evidence will not be provided for an indeterminate result as per COSMIN guidelines [28]. Each PROM then received a recommendation (Class A, B or C) [26]. Class A PROMs are recommended for use and the results within these measures can be trusted due to sufficient content validity and low-quality evidence for sufficient internal consistency. Class B PROMs have the potential to be recommended for use but require further research to assess their quality. Class C PROMs are not recommended for use with high-quality evidence for insufficient psychometric properties.

## Results

**Fig. 1:**
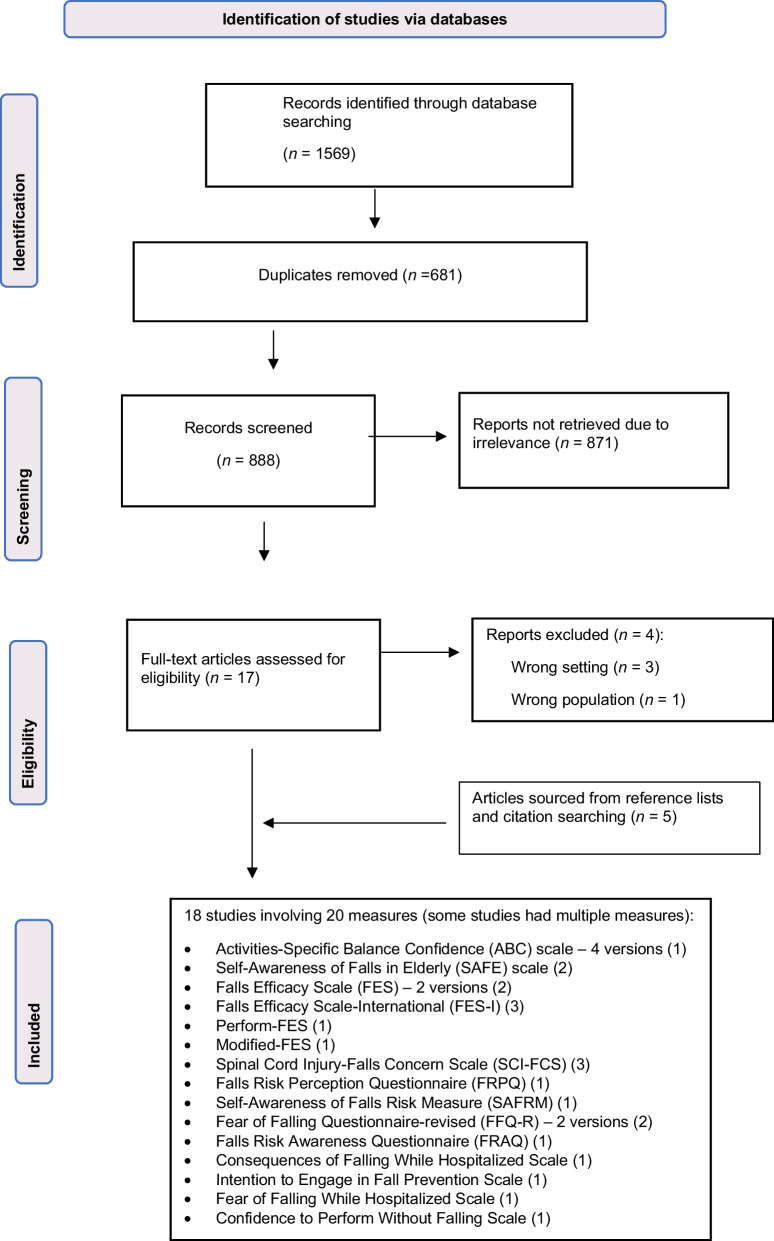
Modified PRISMA flowchart of search strategy [1]

From the initial database search, a total of 1569 citations were identified and uploaded into Covidence. A PRISMA flow chart of the systematic search strategy is shown in Fig. 1, in which 17 full-text studies were obtained and assessed for eligibility. Three papers were excluded because they occurred in outpatient settings and another due to wrong target population (registered nurses). Five additional studies were sourced from reference lists and citation searching, resulting in a total of 18 studies that met the eligibility criteria.

### Article characteristics

From the 18 studies, there was a combined total of 3180 participants with an average age of 70.35 years. A total of 20 fall risk perceptions measures were identified, with an additional five single-item falls-related questions within the 18 studies. The authors collectively agreed to exclude the single-item scales due to insufficient information about their content validity and psychometric properties. Ten of the resulting studies pertained to the development of a PROM. Table 1 displays a data summary table of the 18 included studies and ensuing PROMs.

**Table 1:** Data summary table

References and country Year of tool development and author (if applicable)	Primary fall perception measure and construct(s) measured	Target population and cognitive status of population	Setting	No. of scale items	Description of scale	Interpretation of scoring	Test completion time	Recall period
Birgili et al. [33] and Turkey Original author of SAFE scale: Shyu et al. [20]	Adapted Turkish version of the Self-Awareness of Falls in Elderly (SAFE) scale Construct: Falls Risk Awareness	n = 346 inpatients aged 65 years and older Cognitively intact	Hospital	21-items (four subfactors measuring awareness of activity safety and environment, awareness of physical functions, awareness of medication, awareness of cognitive behaviour)	A 5-pt Likert scale was used to rate awareness levels from 1 = strongly agree to 5 = strongly disagree	Higher scores indicate a high level of falls risk awareness (Score range from 21 to 105)	30 min (all data tools)	Undefined
^1^Bower et al. [34] and USA N/A – developed by author based on the original FFQ by Dayhoff et al. [16]	Fear of Falling Questionnaire-revised (FFQ-R) Construct: Fear of falling	n = 405 adults following a hip fracture, mean age 78.0 years ± 8.7 Cognitively intact or mild cognitive impairment	Hospital/rehabilitation facility	15-item (6-item version proposed in post-hoc analysis)	Likert type scale from 1 to 4, in which patients’ rate whether they strongly disagree, disagree, agree or strongly agree	Higher scores indicate higher levels of FoF (Score range from 15 to 60 for 15-item)	Not reported	Undefined
Büla et al. [35] and Switzerland Original authors: Tinetti et al. [15] later revised to Tinetti et al. [36]	Adapted version of the Falls Efficacy Scale (FES) Construct: Confidence that a person has in performing ADLs without falling	n = 70 adults mean age 81.1 years ± 8.6 Inclusion criteria for MMSE: ≥ 20	Post-acute rehabilitation facility	12-items	Scores for individual ADLs are on a scale ranging from 0 = no confidence to 10 = full confidence	High scores indicate higher confidence in performing ADLs (Score range from 0 to 120)	Median of 4 min, 10 s	Undefined
Caronni et al. [37] and Italy Original author of FES-I: Yardley et al. [38]	Validated Italian version of FES-I Construct: Concern for falling	n = 251 adults with balance impairment, mean age 74 years Cognitively intact	Inpatient rehabilitation	16-items	Scores for individual activities ranged from 1 (not at all concerned) to 4 (very concerned)	Higher scores indicate high concerns about falling (Score range from 16 to 64)	Not reported	Undefined
Choi et al. [21] and South Korea N/A—developed by author	Falls Risk Perception Questionnaire (FRPQ) Construct: Perceived risk of falling	n = 236 mean age 62.2 years ± 10.99 Cognitively intact	Acute care hospitals	27-items (three subfactors measuring personal-mobility, personal-chronic condition and environmental factor)	Scores for statements range from 0 = absolutely not true to 3 = absolutely true	High scores indicate a perception of a high risk for falling (Score range from 0 to 81)	7–15 min to complete both questionnaires (KFES-I and FRPQ)	Undefined
Dautel et al. [39] and Germany Original author of FFQ-R: Bower et al. [34]	German version of the Fear of Falling Questionnaire-revised (FFQ-R) Construct: Fear of falling	n = 152 patients with hip or pelvic fracture, mean age 84.3 years ± 6.2 Inclusion criteria for MMSE: ≥ 24	Inpatient rehabilitation	6-item version	Likert type scale from 1 to 4, in which patients’ rate whether they strongly disagree, disagree, agree or strongly agree	Higher scores indicate higher levels of FOF (Score range from 6 to 24)	Not reported	Undefined
Ferrer Soler et al. [40] and Switzerland N/A—adapted by author based on the Short FES-I scale	Perform-FES Construct: Fear of falling based on performance	n = 52 adults with mean age 85.3 years ± 6.0 Mean MMSE scores = 23.0 ± 4.6 Inclusion criteria for MMSE: not stated	Geriatric hospital	7-items	Scores for individual activities ranged from 1 (not at all concerned) to 4 (very concerned)	Higher scores indicate high concern about falling (Score range from 7 to 28)	15–25 min	Undefined
Franchignoni et al. [41] and Italy Original author of ABC: Powell and Myers [17] Author of ABC-6: Peretz et al. [42] Author of ABC-6: Oude Nijhuis et al. [43] Author of ABC-5: Lohnes and Earhart [44]	Activities-Specific Balance Confidence Scale (ABC) Construct: Confidence in performing an activity without losing balance ABC-6P ABC-6ON ABC-5L	n = 217 patients with PD, mean age 71 years Inclusion criteria for MMSE: ≥ 24	Rehabilitation institute	16-items 6-items 6-items 5-items	Each item rated on a scale from 0% (no confidence) to 100% (full confidence in performing the activity without losing balance) Items 5, 6, 13, 14, 15, 16 Items 5, 6, 12, 13, 15, 16 Items 5, 6, 13, 15, 16	The total score is the mean sum of individual items, where a high score indicates high confidence	Not reported	Undefined Undefined Undefined Undefined
Galante-Maia et al. [45] and Brazil Original author of SCI-FCS: Boswell-Ruys et al. [18]	Brazilian-Portugese version of the Spinal Cord Injury-Falls Concern Scale (SCI-FCS) Construct: Self-report concern about falling	n = 130 adults with SCI, mean age 36 years for pre-test group, 37 years for test–retest group Cognitively intact	Rehabilitation hospitals	16-items	Scores for individual activities ranged from 1 (not at all concerned) to 4 (very concerned)	Higher scores indicate a high concern about falling (Score range from 16 to 64)	Not reported	Undefined
Hauer et al. [46] and Germany Original author of FES: Tinetti et al. [15] later revised to Tinetti et al. [36] Original author of FES-I: Yardley et al. [38]	FES Construct: Confidence that a person has in performing ADLs without falling FES-I Construct: Concern for falling	n = 156 adults mean age 81.7 years ± 6.1 MMSE scores: 24.2 ± 3.7 Inclusion criteria for MMSE: ≥ 17	Geriatric rehabilitation	10-items 16-items	Authors reported that FES data was collected from the first 10 items of the FES-I score, in which scores ranged from 1 (not at all concerned) to 4 (very concerned) – the original scale for FES is scored from 0 = no confidence to 10 = full confidence Scores for individual activities ranged from 1 (not at all concerned) to 4 (very concerned)	High scores indicate high concern about falling (Score range from 10 to 100) according to original scale [15] High scores indicate high concern about falling (Authors state score range from 16 to 56, however original scale is 16–64)	For cognitively impaired: self-report 5.65 min and interview 5.89 min For cognitively intact: self-report 5.65 min and interview 5.89 min	Undefined Undefined
Mihaljcic et al. [19] and Australia N/A—developed by author	Self-Awareness of Falls Risk Measure (SAFRM) Construct: Falls Risk Awareness	n = 91 adults mean age 77.97 years ± 8.04 Inclusion criteria for MMSE: ≥ 18	Inpatient rehabilitation	31-items (three subsections measuring intellectual, emergent and anticipatory self-awareness)	First subsection (intellectual awareness) is measured on a 5pt Likert scale where 1 = greatly increased risk of falling to 5 = no difficulties. Second subsection (emergent awareness) is measured on a 5-pt Likert scale where 1 = much worse to 5 = much better. Third subsection (anticipatory awareness) is measured on a 7-pt Likert scale where 1 = unable to complete, to 7 = complete independence	Awareness scores for each section are calculated by subtracting the clinician ratings from the patient ratings. Larger scores indicate disparities in falls risk awareness. Positive scores demonstrate underestimating falls risk by the patient, negative scores indicate overestimation of falls risk and a score of 0 indicates agreement	10 min for clinician version and 20 min for the patient version	None (current level)
^1^Perrot et al. [47] and France Original author of M-FES: Hill et al. [48]	French version of the Modified-Falls Efficacy Scale (M-FES Fr) Construct: Fear of falling (as described by authors)	n = 56 geriatric patients with mean age of 79.5 years ± 7.6 Cognitive status not reported	Geriatric hospitals	14-items (9-items for indoor activities, 5-items for outdoor activities)	Scores for activities are on a scale ranging from 0 = not at all confident to 10 = completely confident	Higher scores indicate higher confidence in completing activities without falling (Score range from 0 to 140)	Not reported	Undefined
Pramodhyakul and Pramodhyakul [49] and Thailand Original author of SCI-FCS: Boswell-Ruys et al. [18]	Thai version of the Spinal Cord Injury-Falls Concern Scale (SCI-FCS) Construct: Self-report concern about falling	n = 54 adults with SCI with mean age of 31.8 years ± 9.5 Cognitive status not reported	Tertiary rehabilitation setting	16-items	Scores for individual activities ranged from 1 (not at all concerned) to 4 (very concerned)	Higher scores indicate a high concern about falling (Score range from 16 to 64)	Not reported	Undefined
Roaldsen et al. [50] Original author of SCI-FCS: Boswell-Ruys et al. [18]	Norwegian version of the Spinal Cord Injury-Falls Concern Scale (SCI-FCS) Construct: Self-report concern about falling for people with a SCI	n = 56 adults with complete or incomplete SCI with mean age of 49 years Cognitive status not reported	Inpatient rehabilitation setting	16-items	Scores for individual activities ranged from 1 (not at all concerned) to 4 (very concerned)	Higher scores indicate a high concern about falling (Score range from 16 to 64)	5–20 min	Undefined
Shyu et al. [20] and Taiwan N/A—developed by author	SAFE Construct: Falls Risk Awareness	n = 600 adults mean age 70.22 years ± 14.83 Cognitively intact	Inpatients from three medical university hospitals	21-items (four subfactors measuring awareness of activity safety and environment, awareness of physical functions, awareness of medication, awareness of cognitive behaviour)	A 5-pt Likert scale was used to rate awareness levels from 1 = strongly agree to 5 = strongly disagree	Higher scores indicate a high level of falls risk awareness (Score range from 21 to 105)	30-min face to face interview	Undefined
Twibell et al. [51] and USA N/A—developed by author	Confidence to Perform Without Falling Scale Fear of Falling While Hospitalized Scale Consequences of Falling While Hospitalized Scale Intention to Engage in Fall Prevention Scale Perceived Likelihood of Falling While Hospitalized Perceived likelihood of injury if they did fall while hospitalized Perceived fear of falling	n = 158 adults mean age 69.9 years ± 13.37 Cognitively intact	Acute care units	7-items 7-items 12-items 9-items Single item Single item Single item	5-pt Likert scale from 1 = strongly disagree to 5 = strongly agree 4-pt Likert scale from 1 = not at all concerned to 4 = very concerned 4-pt Likert scale from 1 = strongly disagree to 4 = strongly agree 5-pt Likert scale from 1 = strongly disagree to 5 = strongly agree 5-pt Likert scale from 1 = not at all likely to 5 = very likely 5-pt Likert scale from 1 = not at all likely to 5 = very likely 5-pt Likert scale from 1 = not at all likely to 5 = very likely	Higher scores indicate higher confidence (Score range from 7 to 35) Higher scores indicate higher concern (Score range from 7 to 28) Higher scores indicate higher awareness of consequences (Score range from 7 to 28) Higher scores show increased intention to engage (Score range from 9 to 45) As per scale title As per scale title As per scale title	Not reported	Undefined Undefined Undefined Undefined Undefined Undefined Undefined
^1^Visschedijk et al. [52] and the Netherlands Original author of FES-I: Yardley et al. [38]	FES-I Construct: Concern for falling	Group 1, n = 100 adults following a hip fracture, mean age 83.1 years ± 8.3 19% had short-term memory impairment 6% had long-term memory impairment	Skilled nursing facility	16-items	Scores for individual activities ranged from 1 (not at all concerned) to 4 (very concerned)	High scores indicate high concern about falling (Score range from 16 to 64)	3–4 min	Undefined
^1^Wiens et al. [53] and Canada N/A—developed by author	Falls Risk Awareness Questionnaire (FRAQ) Construct: Awareness and perception of risk factors for falling	n = 50 hospitalised respondents, mean age 80 years ± 7.4 Cognitive status not reported, however one person was noted to have Alzheimer’s disease	Acute care and rehabilitation (two separate hospitals)	19-multiple choice questions	Multiple choice questions to assess awareness or perception of select characteristics	Answers were weighted, providing a maximum total score of 24 points, in which a higher score indicates higher awareness of risk factors	15 min	Undefined

*ABC* activities-specific balance confidence scale, *ADLs* activities of daily living, *FES* falls efficacy scale, *FES-I* falls efficacy scale-international, *FFQ-R* fear of falling questionnaire-revised, *FoF* fear of falling, *FRAQ* falls risk awareness questionnaire, *FRPQ* falls risk perception questionnaire (FRPQ), *MMSE* mini-mental state examination, *PD* Parkinson’s disease, *SAFE* self-awareness of falls in elderly (SAFE) scale, *SAFRM* self-awareness of falls risk measure, *SCI* spinal cord injury, *SCI-FCS* spinal cord injury-falls concern scale

^1^Study population was mixed in which we included only the inpatient population for this study

### Fall-related constructs

Given the diversity of the constructs featured in the falls risk perception instruments, the authors formatted the tabulated findings according to five fall-related constructs: Balance Confidence, Fall-related Self-Efficacy, Fear of Falling, Falls Risk Awareness and Outcome Expectancy. These were classified based on previous research of fall-related psychological constructs by Moore and Ellis [24] and Hughes et al. [54], with the exception of Falls Risk Awareness. Prior research has shown that fall-related constructs are comparable and often used interchangeably, which is why researchers are encouraged to classify the constructs being measured to avoid confusion [24, 54]. For example, Balance Confidence relates to an individual’s belief about their ability to maintain balance whilst performing functional activities [54], whereas Fall-related Self-Efficacy pertains to a person’s confidence to undertake functional activities without falling [15]. Similarly, Fear of Falling refers to a person’s concern about falling, however this is usually associated with avoidance of activities and may include heightened emotional states [36, 54]. The construct of Outcome Expectancy pertains to beliefs about the anticipated consequences of falling [54]. The authors opted to include Falls Risk Awareness as a construct, which draws upon the person’s understanding of their personal strengths and limitations [55].

Table 2 provides the overall ratings for each PROM using the risk of bias checklist and quality of evidence. Each box in this table contains two ratings, with the exception of those listed as ‘not applicable’ or ‘not reported’. The symbol in the top row of each box pertains to the risk of bias rating, whereas the second row of the box contains the quality of evidence rating for each measurement property.

**Table 2:** Summary of findings and quality ratings

Scale	Targeted population	Content validity	Structural validity	Internal consistency	Cross cultural validity	Reliability	Measurement error	Hypotheses testing for construct validity	Responsiveness	Recommendations using
*Construct: balance confidence*										
Activities-Specific Balance Confidence Scale (ABC) 16 item version	Hospitalised adults with PD	? Not assessed	+ Moderate	+ High	N/A	+ High	NR	+ High	NR	B
ABC-6P [42]	Hospitalised adults with PD	? Not assessed	+ Moderate	+ High	N/A	+ High	NR	+ High	NR	B
ABC-6ON [43]	Hospitalised adults with PD	? Not assessed	+ Moderate	+ High	N/A	+ High	NR	+ High	NR	B
ABC-5L [44]	Hospitalised adults with PD	? Not assessed	+ Moderate	+ High	N/A	+ High	NR	+ High	NR	B
*Construct: fall-related self-efficacy*										
Adapted version of the falls efficacy scale (FES) 12 item version	Older adults in post-acute rehabilitation	? Not assessed	− Low	+ Moderate	N/A	+ Low	NR	+ Moderate	NR	B
Modified-Falls Efficacy Scale (M-FES)	Older hospitalised adults	? Not assessed	+ Moderate	+ Moderate	+ (French) Moderate	+ Moderate	NR	+ Moderate	NR	B
Falls Efficacy Scale (FES) 10 item version	Older adults in geriatric rehabilitation	? Not assessed	NR	+ Moderate	N/A	+ Moderate	NR	− Low	NR	B
Falls Efficacy Scale—International (FES-I)	Older adults in geriatric rehabilitation	? Not assessed	± Moderate	+ High	N/A	± Moderate	± Moderate	± Moderate	NR	B
Perform-FES	Older hospitalised adults	? Not assessed	NR	+ Moderate	N/A	+ Low	NR	+ Moderate	NR	B
Spinal Cord Injury-Falls Concern Scale (SCI-FCS)	Adults with a spinal cord injury	+ High	± Moderate	+ Moderate	± (Brazilian- Portugese, Norwegian, Thai) Moderate	+ Moderate	± Moderate	− Moderate	NR	A
Confidence to Perform Without Falling Scale	Older adults in acute care units	? Not assessed	− Low	+ Moderate	N/A	− Low	NR	− Low	NR	C
*Construct: Fear of Falling*										
Fear of Falling Questionnaire-revised (FFQ-R) 15 items	Older adults following a hip fracture	? Not assessed	+ Moderate	+ High	N/A	+ Moderate	NR	+ High	NR	B
Fear of Falling Questionnaire-revised (FFQ-R) 6 items	Older adults following a hip or pelvic fracture	? Not assessed	+ High	+ High	+ (German)	+ High	NR	+ High	NR	B
Fear of Falling While Hospitalized Scale	Older adults in acute care units	? Not assessed	− Low	+ Moderate	N/A	− Low	NR	− Low	NR	C
*Construct: falls risk awareness*										
Self-Awareness of Falls in Elderly (SAFE) scale	Older hospitalised adults	? Not assessed	+ High	+ High	+ (Turkish) High	+ High	NR	+ High	NR	B
Self-Awareness of Falls Risk Measure (SAFRM)	Older adults in inpatient rehabilitation	? Not assessed	+ Moderate	+ Moderate	N/A	+ Moderate	NR	+ Moderate	NR	B
Falls Risk Awareness Questionnaire (FRAQ)	Older hospitalised adults	? Not assessed	NR	NR	N/A	NR	NR	− Very low	NR	C
Falls Risk Perception Questionnaire (FRPQ)	Older hospitalised adults	+ High	+ Moderate	+ High	N/A	− Low	NR	+ High	NR	A
*Construct: outcome expectancy*										
Consequences of Falling While Hospitalized Scale	Older adults in acute care units	? Not assessed	− Low	+ Moderate	N/A	− Low	NR	− Low	NR	C
Intention to Engage in Fall Prevention Scale	Older adults in acute care units	? Not assessed	− Low	+ Moderate	N/A	− Low	NR	− Low	NR	C

When results were indeterminate (?), not reported (NR) or not applicable (N/A), the overall quality of evidence rating was not assessed

+ : Sufficient: ≥ 85% of the items of the PROM (or subscale) fulfill the criteria

?: Indeterminate: Not enough information or no information available or quality of the study is inadequate

± : Inconsistent

− : Insufficient: < 85% of the items of the PROM (or subscale) does fulfill the criteria

N/A: Not applicable

NR: Not reported

A: Sufficient content validity and at least low-quality evidence for internal consistency

B: PROMs that are neither A nor C

C: insufficient psychometric properties

*ABC* activities-specific balance confidence scale, *ADLs* activities of daily living, *CV* content validity, *CVI* content validity index, *FES* falls efficacy scale, *FES-I* falls efficacy scale-international, *FFQ-R* fear of falling questionnaire-revised, *FoF* fear of falling, *FRAQ* falls risk awareness questionnaire, *FRPQ* falls risk perception questionnaire (FRPQ), *MMSE* mini-mental state examination, *PD* Parkinson’s disease, *SAFE* self-awareness of falls in elderly (SAFE) scale, *SAFRM* self-awareness of falls risk measure, *SCI* spinal cord injury, *SCI-FCS* spinal cord injury-falls concern scale

### Content validity

According to recommendations, content validity should be rated as indeterminate if there is uncertainty of what has been done [30]. Therefore, the content validity of most PROMs was rated as indeterminate as it was unclear whether patients were consulted on comprehensiveness and comprehensibility of the measure during PROM development. Additional file 3 contains the results of the ratings of each PROM for content validity. Only two PROMs were rated as having sufficient content validity (falls risk perception questionnaire [FRPQ] and the spinal cord injury-falls concern scale [SCI-FCS]).

### Psychometric assessments and quality of evidence

Each individual PROM was assessed for structural validity, internal consistency, reliability, cross-cultural validity, measurement error and hypothesis testing for construct validity. These are displayed in additional files 4 and 5. No studies reported responsiveness and only two PROMs featured measurement error (SCI-FCS and the Falls Efficacy Scale-International [FES-I]).

### Balance confidence measures

Four versions of the activities-specific balance confidence (ABC) scale were reviewed in one study for inpatients with Parkinson’s disease [41]. Both classical test theory (CTT) and Rasch analysis was used to analyse the psychometric properties of the four PROMs, with the 16-item version demonstrating higher internal consistency and reliability compared to the shorter scales. The measurement properties of the ABC were improved when a five-level response format (0 = no confidence, 1 = low confidence, 2 = moderate confidence, 3 = high confidence, 5 = complete confidence) was rescaled instead of the usual eleven-level rating scale (0% = no confidence to 100% = full confidence).

### Fall-related self-efficacy measures

Falls-related self-efficacy measures featured the greatest number of PROMs (*n* = 7), compared to other fall-related constructs. The FES-I featured in three studies [37, 46, 52], with inconsistent results for most measurement properties, except for high internal consistency. Results for risk of bias assessments for both the adapted version of the falls efficacy scale (FES) and the modified-FES (MFES) were also downgraded due to imprecision (sample size < 100).

Concern for falling for those with spinal cord injuries also featured through three translated validity studies of the SCI-FCS [45, 49, 50]. Although these studies contained a mixture of moderate to high levels of evidence of psychometric properties, the SCI-FCS has sufficient content validity and internal consistency.

### Fear of falling measures

Although the content validity was considered indeterminate, the pooled results of the 6-item fear of falling questionnaire-revised (FFQ-R) demonstrated high-levels of evidence for psychometric properties and is validated in both English [34] and German [39]. The two-factor item structure (degree of threat and harm outcomes) provides a valid and reliable assessment of fear of falling in hospital. Compared to its 15-item counterpart, the 6-item version is more feasible, however both versions are limited to older adults with hip or pelvic fractures in hospital.

### Falls risk awareness measures

Self-awareness of falls risk was measured in four PROMs, with three of these measures assessed as moderate and high levels of evidence. The Self-Awareness of Falls in Elderly (SAFE) scale was assessed as high-quality evidence for structural validity, internal consistency and reliability from two studies [20, 33]. This scale has been evaluated in Turkish and evaluates perceived awareness of activity safety and environment, awareness of physical functions, awareness of medication and awareness of cognitive behaviour in elderly patients in hospital [20]. The self-awareness of falls risk measure (SAFRM) was downgraded to a moderate rating because of imprecision (sample size < 100), however this was the only instrument to measure both the patient and clinician perception in a rehabilitation setting. Similarly, the falls risk perception questionnaire (FRPQ) shows promise with sufficient content validity and high internal consistency (α = 0.948) in an acute care setting.

### Outcome expectancy measures

Only two PROMs with low levels of evidence featured in this category. Although this construct largely pertains to anticipated consequences of falling, the authors included the Intention to Engage in Fall Prevention Scale in this category. Measuring behaviour or intention to participate could be plausibly viewed as an expected outcome. Both the Intention to Engage in Fall Prevention Scale and the Consequences of Falling While Hospitalised Scale reported high internal consistency (α = 0.90 and 0.84 respectively), yet had insufficient information about content validity, structural validity and reliability.

### Feasibility

The PROMs ranged from 5-items to 31-items taking approximately 5–30 min to complete. Administration of the PROMs by health professionals did not require any equipment apart from a pen/pencil and the measure to record answers. Two of the measures (SAFRM and Perform-FES) included a functional assessment, however all of the equipment required for these are traditionally available in rehabilitation settings. Several PROMs have been translated and validated in other languages (FES-I, FES, MFES, FFQ-R, ABC, SAFE, SCI-FCS).

Some PROMs were developed specifically for community-dwelling therefore their relevance to an inpatient setting may be doubtful. For example, in the ABC scale participants with Parkinson’s disease are asked to rate their perceived level of balance confidence when performing common indoor and outdoor activities of daily living. Activities such as “standing on a chair to reach” or “ride an escalator not holding the rail” may not be applicable to an inpatient setting, which highlights the importance of establishing content validity in diverse populations/settings.

### Recommendations

As demonstrated in Table 2, only two PROMs received Class A recommendations for sufficient content validity and internal consistency (FRPQ and SCI-FCS). The SCI-FCS is recommended for use to assess falls concern in populations with spinal cord injuries. The FRPQ is recommended to assess falls risk perception in an acute care setting, however as there was only one study on PROM development for this instrument, further studies may be needed to assess the use of this PROM in other contexts/populations. Many other PROMs received Class B ratings, indicating that further research on the psychometric properties of these measures is warranted.

## Discussion

The overarching aim of this systematic review was to provide a summary of the quality of falls risk perception measures for adults in a hospital setting. Generally, PROMs can be used to detect physical or psychological concerns, facilitate patient-clinician communication, monitor or provide information about the impact of an intervention and monitor outcomes for quality improvement [9]. Given the subjective nature of PROMs, the COSMIN methodology provides a comprehensive evidence-based framework to improve the selection of outcome measurement instruments for clinical practice [26]. The literature search resulted in a total of 20 PROMs that were categorised according to five fall-related constructs: Balance Confidence, Fall-related Self-Efficacy, Fear of Falling, Falls Risk Awareness and Outcome Expectancy. This review has expanded on previous findings by Moore and Ellis [24] and Hughes et al. [54] by proposing the addition of Falls Risk Awareness as a falls-related construct. Only two PROMs (SCI-FCS and FRPQ) received Class A recommendations, from the Falls-related Self-Efficacy and Falls Risk Awareness categories based on the COSMIN criteria. Even so, these PROMs have been validated in specific patient cohorts, which are not generalisable to all populations and/or contexts. Therefore, these Class A recommendations are established on the populations and contexts described in Table 1.

Many of the PROMs were developed prior to the publication of the COSMIN standards, which may explain why patient populations were not included in the original PROM development. Although COSMIN standards were originally developed to evaluate the quality of studies on the psychometric properties of PROMs [26], they could also be used to guide PROM development. Researchers should consider the inclusion of cognitive interviews with the patient population of interest as stakeholders in the development of PROMs. Some of the PROMs such as the ABC and FES-I, were specifically developed for community-dwelling adults. As stated earlier, some of the items in the ABC may not be relevant to a hospital environment. A recommendation from Moore and Ellis [24] is that measures of efficacy should be composed of items specific to the task of interest, rather than using one overarching falls-related psychological measure. The five fall-related constructs in this review, contained 13 PROMs with Class B recommendations, which signifies that more validation studies are needed for these PROMs. Typically, new instruments are developed because validation studies are too slow to appear [24], therefore future studies could investigate these pre-existing PROMs for measuring the intended construct.

Recent World Guidelines for Falls Prevention and Management provided a strong recommendation to use a standardised instrument such as the FES-I or Short FES-I for assessing concerns about falling in acute care hospitals or long-term care facilities [14]. However, these recommendations were based on an unpublished systematic review and meta-analysis about the four variants of the FES-I (paper in preparation). In comparison, a COSMIN review by Soh et al. [25] reported a lack of high quality evidence for falls efficacy-related scales; though this was not specific to a hospital context. ‘Fear of falling’ and ‘falls efficacy’ are often used interchangeably [22], however studies show they are different [56]. Soh et al. [25] proposed that falls efficacy should be considered across a continuum from pre-fall, near-fall, fall-landing and completed fall, providing researchers with the opportunity to develop instruments based on each proposed domain of falls efficacy.

Although PROMs can facilitate a person-centred approach to falls management, clinicians need to consider the purpose of the PROM and the population/context to avoid inappropriate instrument selection. Given the multifactorial nature of falls in hospital, there is no ‘gold standard’ or one single tool that will provide a complete falls risk assessment [57]. A recent systematic review and meta-analysis found that evidence-based falls education can reduce hospital falls rates [13]. The selection of a clinically relevant PROM provides the opportunity for health professionals to engage with the patient and tailor educational strategies according to their needs. Therefore, future studies should evaluate the implementation of falls-related PROMs in a hospital context and their role in informing instrument selection for falls management.

## Limitations

This review featured studies published in the English language only, which may have limited the findings from our search strategy. Although the 20-year date range of the literature search may incur limitations, some of the PROMs in our included studies were developed prior to 2002. This review featured validation studies of PROMs in an inpatient context, thereby excluding falls-perception measures for community-settings.

One particular criticism of the COSMIN process is the reliance on the ability of the authors to review and appraise the quality of the PROMs [58]. Although subjective judgement is necessary for the COSMIN process, the authors remained transparent with this review by providing additional data files and including people with expertise in PROM development and validation. To the best of our knowledge, this is the first review to apply COSMIN methodology to PROMs of various falls-related constructs in a hospital setting.

## Conclusion

This COSMIN systematic review provided an evaluation of contemporary falls-risk perception measures in an inpatient setting. Although two of the PROMs received a Class A recommendation, further research is needed to validate the use of other Class B PROMs in various patient populations. The take-home message from this review is to include populations of interest as stakeholders in PROM development, to ascertain sufficient content validity of the intended construct.

## Supplementary Information

**Additional file 1.** Search strategy.

**Additional file 2.** Definition table of measurement properties.

**Additional file 3.** Content validity.

**Additional file 4.** Measurement properties of included studies.

**Additional file 5.** Further measurement properties of included studies.

## Data Availability

All data generated or analysed during this study are included in this published article (and its supplementary information files).
