# Supplementary material for: Falls risk perception measures in hospital: a COSMIN systematic review
Source: J Patient Rep Outcomes. 2023 Jun 26;7:58. doi: 10.1186/s41687-023-00603-w (PMC10293508; doi:10.1186/s41687-023-00603-w)
Supplement: Supplementary file 1 — Additional file 1. Search strategy. [file 41687_2023_603_MOESM1_ESM.docx]

**Additional file 1: Search strategy**

| **Search strategy** |
| --- |
| S1) (Fall or falls or falling) AND (efficacy OR fear OR percept* OR perceived OR awareness OR attitude* OR confiden*) |
| S2) outcome* OR measur* OR tool* OR scale* OR instrument* OR questionnaire* OR survey* OR index* |
| S3) reliab* OR valid* OR predict* |
| S4) psychometric OR quantitative |
| S5) NOT children OR paediatric OR pediatric or adolesc* or teenager |
| S1) AND S2) AND S3) AND S4) NOT S5) |

**Key**

* = truncated search term
