# Supplementary material for: Falls risk perception measures in hospital: a COSMIN systematic review
Source: J Patient Rep Outcomes. 2023 Jun 26;7:58. doi: 10.1186/s41687-023-00603-w (PMC10293508; doi:10.1186/s41687-023-00603-w)
Supplement: Supplementary file 2 — Additional file 2. Definition table of measurement properties. [file 41687_2023_603_MOESM2_ESM.docx]

| **Measurement property**  Additional file 2: Definition table of measurement properties | **Rating** | **Criteria** |
| --- | --- | --- |
| Structural validity | + | **CTT**  CFA: CFI or TLI or comparable measure >0.95 OR RMSEA <0.06 OR SRMR <0.08 (factor structures should be equal across studies)  **IRT/Rasch**  No violation of unidimensionality (factor analysis per subscale): CFI or TLI or comparable measure > 0.95 OR RMSEA <0.06 OR SRMR <0.08  AND  no violation of local independence: residual correlations among the items after controlling for the  dominant factor < 0.20 OR Q3’s < 0.37  AND  no violation of monotonicity: adequate looking graphs OR item scalability > 0.30  AND  adequate model fit  IRT: χ2 > 0.001  Rasch: infit and outfit mean squares ≥ 0.5 and ≤ 1.5 OR Z-standardized values > −2 and < 2 |
|  | ? | **CTT:** not all information for + reported  **IRT:** model fit not reported |
|  | - | Criteria for + not met |
| Internal consistency | + | Cronbach’s alpha ≥0.70 for each unidimensional scale or subscale AND “At least low evidence” for sufficient structural validity (may come from different studies) |
|  | ? | Criteria for “At least low evidence” for sufficient structural validity not met |
|  | - | Cronbach’s alpha <0.70 for each unidimensional scale or subscale AND “At least low evidence” for sufficient structural validity not met |
| Reliability | + | ICC or weighted Kappa ≥0.70 |
|  | ? | ICC or weighted Kappa not reported |
|  | - | ICC or weighted Kappa <0.70 |
| Measurement Error | + | SDC or LoA < MIC⁵ |
|  | ? | MIC not defined |
|  | - | SDC or LoA > MIC⁵ |
| Hypotheses testing for construct validity | + | The result is in accordance with the hypothesis |
|  | ? | No hypothesis defined (by the review team) |
|  | - | The results are not in accordance with the hypothesis |
| Cross-cultural validity/measurement invariance | + | No important differences between group factors such as age, gender, language in multiple group factor analysis OR no important DIF for group factors (McFadden’s R² <0.02) |
|  | ? | No multiple group factor analysis OR DIF analysis performed |
|  | - | Important differences between group factors or DIF was found |

This criteria guide is adapted from Prinsen et al. (2018)

**Key**:

+ = sufficient

? = indeterminate

- = insufficient

**Abbreviations:** CFA: confirmatory factor analysis; CFI: comparative fit index; CTT: classical test theory; DIF: differential item functioning; ICC: intraclass correlation coefficient; IRT: item response theory; LoA: limits of agreement; MIC: minimal important change; RMSEA root mean square error of approximation; SDC: smallest detectable change; SRMR: standardised root mean residuals; TLI: Tucker-Lewis index

**References:** Prinsen, C. A. C., Mokkink, L. B., Bouter, L. M., Alonso, J., Patrick, D. L., de Vet, H. C. W., & Terwee, C. B. (2018). COSMIN guideline for systematic reviews of patient-reported outcome measures. *Quality of life research : an international journal of quality of life aspects of treatment, care and rehabilitation*, *27*(5), 1147-1157. <https://doi.org/10.1007/s11136-018-1798-3>
