# Supplementary material for: Falls risk perception measures in hospital: a COSMIN systematic review
Source: J Patient Rep Outcomes. 2023 Jun 26;7:58. doi: 10.1186/s41687-023-00603-w (PMC10293508; doi:10.1186/s41687-023-00603-w)
Supplement: Supplementary file 3 — Additional file 3. Content validity. [file 41687_2023_603_MOESM3_ESM.docx]

| **Scale**  **Additional file 3: Content Validity** | **Scale or Pooled Result** | **Type of Measurement Instrument** | **Relevance**  **Rating Comments** | | **Comprehensiveness**  **Rating Comments** | | **Comprehensibility**  **Rating Comments** | |
| --- | --- | --- | --- | --- | --- | --- | --- | --- |
| **Construct: Balance Confidence** | | | | | | | | |
| Activities-Specific Balance Confidence Scale (ABC) 16 item version | Pooled result (1 CV and in consultation with systematic review, Soh et al 2021) | PROMs | ? | Insufficient information about concept elicitation (Soh et al., 2021) | ? | Unclear if patients were asked about comprehensiveness of measure | ? | Unclear if patients were asked about comprehensibility of the 6-item measure |
| ABC-6P (Peretz et al., 2006) | Pooled result (1 CV with consultation of the original PROM development) | PROMs | + | Construct of interest is sufficiently described as per COSMIN guidelines | ? | Unclear if patients were asked about comprehensiveness of measure during concept elicitation of the 6-item scale | ? | Unclear if patients were asked about comprehensibility of the 6-item measure |
| ABC-6ON (Oude Nijuis et al., 2007) | Pooled result (consultation from previous systematic review, Soh et al 2021) | PROMs | ? | Construct of interest is sufficiently described as per COSMIN guidelines | ? | Unclear if patients were asked about comprehensiveness of measure | ? | Unclear if patients were asked about comprehensibility of the 6-item measure |
| ABC-5L (Lohnes and Earhart, 2010) | Pooled result (1 CV with consultation of the original PROM development) | PROMs | ? | Construct of interest is sufficiently described as per COSMIN guidelines | ? | Unclear if patients were asked about comprehensiveness of measure | ? | Unclear if patients were asked about comprehensibility of the 6-item measure |
| **Construct: Fall-Related Self-Efficacy** | | | | | | | | |
| Adapted version of the Falls Efficacy Scale (FES) – 12 items | One study | PROMs | + | Construct of interest is sufficiently described as per COSMIN guidelines | ? | Unclear if patients were asked about comprehensiveness | ? | Unclear if patients were asked about comprehensibility of measure |
| Modified-Falls Efficacy Scale (M-FES) | Pooled result (1 CV and in consultation with systematic review, Soh et al 2021) | PROMs | + | Construct of interest is sufficiently described as per COSMIN guidelines | ? | Unclear if patients were asked about comprehensiveness | ? | Unclear if patients were asked about comprehensibility of measure |
| Falls Efficacy Scale (FES) – 10 items | Pooled result (1 CV and in consultation with systematic review, Soh et al 2021) | PROMs | + | Construct of interest is sufficiently described as per COSMIN guidelines | ? | Unclear if patients were asked about comprehensiveness | ? | Unclear if patients were asked about comprehensibility of measure |
| Falls Efficacy Scale – International (FES-I) | Pooled result (3 CV and in consultation with systematic review, Soh et al 2021) | PROMs | + | Construct of interest is sufficiently described as per COSMIN guidelines | ? | Unclear if patients were asked about comprehensiveness | ? | Unclear if patients were asked about comprehensibility of measure |
| Perform-FES | One study | PerFOMS/ PROMs | + | Construct of interest is sufficiently described as per COSMIN guidelines | ? | Unclear if patients were asked about comprehensiveness | ? | Unclear if patients were asked about comprehensibility of measure |
| Spinal Cord Injury-Falls Concern Scale (SCI-FCS) | Pooled result (3 CV with consultation of the original PROM development Boswell-Ruys et al., 2010) | PROMs | + | Construct of interest is sufficiently described as per COSMIN guidelines | ? | Scale was developed by a total of 22 health professionals. Unclear if patients were asked about comprehensiveness of measure during concept elicitation | + | Target population was asked about comprehensiveness of measure |
| Confidence to Perform Without Falling Scale | One study | PROMs | ? | Not enough information about concept elicitation phase | ? | Insufficient information about pilot testing and expert panel review | ? | Insufficient information about pilot testing and expert panel review |
| **Construct: Fear of Falling** | | | | | | | | |
| Fear of Falling Questionnaire-revised (FFQ-R) 15-item | One study | PROMs | + | Construct of interest is sufficiently described as per COSMIN guidelines | ? | Unclear if patients were asked about comprehensiveness | ? | Revisions made by clinicians who are experts in geriatric health, no information about patient involvement |
| Fear of Falling Questionnaire-revised (FFQ-R) 6-item | Pooled result (1 PROM development & 1 CV) | PROMs | ? | Developed through post hoc analysis – insufficient information about concept elicitation | ? | Unclear if patients were asked about comprehensiveness of measure during concept elicitation | ? | Unclear if patients were asked about comprehensibility of measure |
| Fear of Falling While Hospitalized Scale | One study | PROMs | ? | Not enough information about concept elicitation phase | ? | Insufficient information about pilot testing and expert panel review | ? | Insufficient information about pilot testing and expert panel review |
| **Construct: Falls Risk Awareness** | | | | | | | | |
| Self-Awareness of Falls in Elderly (SAFE) scale | Pooled result (1 PROM development & 1 CV) | PROMs | + | Construct of interest is sufficiently described as per COSMIN guidelines | ? | CVI established by 15 experts in total.  Unclear if patients were asked about comprehensiveness of measure during concept elicitation | ? | Unclear if patients were asked about comprehensibility of measure |
| Self-Awareness of Falls Risk Measure (SAFRM) | One study | PROMs/ ClinROMs | + | Construct of interest is sufficiently described as per COSMIN guidelines | ? | Unclear if patients were asked about comprehensiveness of measure during concept elicitation | ? | Unclear if patients were asked about comprehensibility of measure |
| Falls Risk Awareness Questionnaire (FRAQ) | One study | PROMs | ? | Not enough information about concept elicitation phase | ? | Insufficient information about comprehensiveness of measure | + | Minor wording revisions were made based on participant and expert feedback |
| Falls Risk Perception Questionnaire (FRPQ) | One study | PROMs | + | Construct of interest is sufficiently described as per COSMIN guidelines | + | CVI established by 9 experts in two rounds. Preliminary items were based on interviews from patients who had fallen | + | 20 inpatients interviewed to determine comprehensibility |
| **Construct: Outcome Expectancy** | | | | | | | | |
| Consequences of Falling While Hospitalized Scale | One study | PROMs | ? | Not enough information about concept elicitation phase | ? | Insufficient information about pilot testing and expert panel review | ? | Insufficient information about pilot testing and expert panel review |
| Intention to Engage in Fall Prevention Scale | One study | PROMs | ? | Not enough information about concept elicitation phase | ? | Insufficient information about pilot testing and expert panel review | ? | Insufficient information about pilot testing and expert panel review |

**Key:**

**+** Sufficient: ≥85% of the items of the PROM (or subscale) fulfill the criteria

**?** Indeterminate: Not enough information or no information available or quality of the study is inadequate

**-** Insufficient: <85% of the items of the PROM (or subscale) does fulfill the criteria

**Abbreviations:**

ABC: Activities-Specific Balance Confidence Scale; ADLs: Activities of Daily Living; CV: Content validity; CVI: Content Validity Index; FES: Falls Efficacy Scale; FES-I: Falls Efficacy Scale – International; FFQ-R: Fear of Falling Questionnaire-revised; FoF: Fear of Falling; FRAQ: Falls Risk Awareness Questionnaire; FRPQ: Falls Risk Perception Questionnaire (FRPQ); MMSE: Mini-Mental State Examination; PD: Parkinson’s Disease; SAFE: Self-Awareness of Falls in Elderly (SAFE) Scale; SAFRM: Self-Awareness of Falls Risk Measure; SCI: Spinal Cord Injury; SCI-FCS: Spinal Cord Injury-Falls Concern Scale

**References:**

1. Terwee CB, Prinsen C, Chiarotto A, De Vet H, Bouter LM, Alonso J, et al. COSMIN methodology for assessing the content validity of PROMs–user manual. Amsterdam: VU University Medical Center. 2018.
