# Supplementary material for: Falls risk perception measures in hospital: a COSMIN systematic review
Source: J Patient Rep Outcomes. 2023 Jun 26;7:58. doi: 10.1186/s41687-023-00603-w (PMC10293508; doi:10.1186/s41687-023-00603-w)
Supplement: Supplementary file 4 — Additional file 4. Measurement properties of included studies. [file 41687_2023_603_MOESM4_ESM.docx]

| **Scale & Author**  **Additional file 4: Measurement properties of included studies** | **Structural validity**  **COSMIN**  **Rating Analysis Model Comments** | | | **Internal Consistency**  **COSMIN**  **Rating Comments** | | **Reliability**  **COSMIN**  **Rating Comments** | |
| --- | --- | --- | --- | --- | --- | --- | --- |
| **Construct: Balance Confidence** | | | | | | | |
| Activities-Specific Balance Confidence Scale (ABC) (Franchignoni et al., 2014) | Adequate | EFA, PCA & Rasch analysis | 64% of variance explained by the Rasch factor, eigenvalue = 2.2 | Very good | **Cronbach’s alpha** = .95 (CTT) | Very good | Evaluated via person separation reliability, which is an index similar to Cronbach’s alpha  ABC = 0.93 (Rasch) |
| ABC-6P | Adequate | EFA, PCA & Rasch analysis | 68.1% of variance explained by the Rasch factor, eigenvalue = 1.4 | Very good | **Cronbach’s alpha** = .89 (CTT) | Very good | ABC-6P = 0.84 (Rasch) |
| ABC-6ON | Adequate | EFA, PCA & Rasch analysis | 70.6% of variance explained by the Rasch factor, eigenvalue = 1.5 | Very good | **Cronbach’s alpha** = .90 (CTT) | Very good | ABC-6ON = 0.85 (Rasch) |
| ABC-5L | Adequate | EFA, PCA & Rasch analysis | 70.9% of variance explained by the Rasch factor, eigenvalue = 1.5 | Very good | **Cronbach’s alpha** = .88 (CTT) | Very good | ABC-5L = 0.82 (Rasch) |
| **Construct: Fall-Related Self-Efficacy** | | | | | | | |
| Adapted version of the Falls Efficacy Scale (FES)  (Bula et al., 2008) | Doubtful (sample size not calculated) | Factor analysis using PCA with varimax rotation | Factor analysis suggested a 2-factor solution accounting for 63.7% of the variance | Very good | **Cronbach’s alpha** = .90 | Very good | ICC=0.97  Test-retest period 3 days |
| Modified-Falls Efficacy Scale (M-FES) (Perrot et al., 2018) | Adequate | Factor analysis with oblique rotation | Kaiser-Meyer-Olkin = 0.93  Two factor solution was suggested which accounted for 68.1% of the total variance after using the maximum likelihood method, the scree test and the eigenvalue > 1 rule. | Very good | Cronbach’s alpha scores  Total = 0.96  Indoor subscale: 0.94  Outdoor subscale: 0.94 | Adequate | ICC = 0.96  Test-retest period 7 days  Indoor subscale: ICC = 0.93  (*p* < 0.01)  Outdoor subscale: ICC = 0.96 (*p* < 0.01) |
| Falls Efficacy Scale (FES) (Hauer et al., 2010) | Inadequate | Not reported | Not reported | Very good | Cronbach’s alpha scores ranged from 0.890-0.923 for both cognitively impaired and cognitively intact groups | Very good | ICC ranged from 0.81 to 0.88  Test-retest 5-7 days |
| Falls Efficacy Scale – International (FES-I) (Caronni et al., 2021) | Very good | Rasch analysis – RSM (Rating scale model) – results show the FES-I is properly ordered | Infit mean square statistic ranging from 0.8 to 1.32 & Outfit mean square ranging from 0.71 to 1.45  Which indicates a good model fit (0.5-1.5) | Very good | Reliability: 0.87 allowing to distinguish 3 to 4 H strata (the FES-I can grade the concern about falling on up to 4 levels)  Rasch reliability corresponds to Cronbach’s alpha | Inadequate | Reliability of 0.87 reported as internal consistency |
| Falls Efficacy Scale – International (FES-I) (Hauer et al., 2010) | Inadequate | Not reported | Not reported | Very good | **Cronbach’s alpha** scores ranged from 0.925-0.957 for both cognitively impaired and cognitively intact groups | Very good | ICC ranged from 0.58 to 0.92  Results were lower for the group with cognitive impairment (0.58) as expected  Test-retest 5-7 days |
| Falls Efficacy Scale – International (FES-I) (Visschedijk et al., 2015) | Adequate | CFA for categorical items with factor loadings >0.50 | One factor model fitted the data with CFI 0.994 and RMSEA 0.047 | Very good | **Cronbach’s alpha** = 0.94 | Very good | ICC for all raters was 0.72 |
| Perform-FES  (Ferrer Soler et al., 2021) | Inadequate | Not reported – scale derived from the Short FES-I | Not reported | Very good | **Cronbach’s alpha** = 0.78 | Very good | **ICC = 0**.94  Test-retest period: 2 days  AUC was 0.81 for PERFORM-FES compared with 0.61 (FES-I), 0.68 (ABC-5), 0.56 (Short FES-I), 0.65 (GFFM) with reference of 0.50 (no better than chance alone) |
| Spinal Cord Injury-Falls Concern Scale (SCI-FCS)  (Galante-Maia et al., 2021) | Adequate | Rasch Analysis (Rating scale model) & PCA | Infit & Outfit mean squares >0.5 and <1.5  53% of the total variance was explained by the principal component | Very good | **Cronbach’s alpha** = 0.95 | Very good | ICC = 0.92  Test-retest period 3-7 days  Kappa co-efficients ranged from 0.04 to 0.87 of individual items |
| Spinal Cord Injury-Falls Concern Scale (SCI-FCS)  (Pramodhyakul & Pramodhyakul, 2019) | Inadequate | Not reported – process of translation from English to Thai described | Not reported | Very good | **Cronbach’s alpha =** 0.88 | Very good | ICC = 0.99 *p* < 0.001  Test-retest period 7 days |
| Spinal Cord Injury-Falls Concern Scale (SCI-FCS)  (Roaldsen et al., 2016) | Inadequate | Not reported – process of translation from English to Norwegian described | Not reported | Very good | **Cronbach’s alpha =** 0.88 | Very good | ICC = 0.83  Test-retest period: twice within one week  PA (≥70%) was noted for all 16 items except for item 12  SEM = 2.6  SDC = 7.1 |
| Confidence to Perform Without Falling Scale (Twibell et al., 2015) | Doubtful | Principal axis factor analysis | 73% of variance explained, eigenvalue = 5.1 (no mention of scree plot or of what was used to determine acceptable variance) | Very good | **Cronbach alpha** = 0.94 | Inadequate | Not reported – Cronbach’s alpha scores were reported as internal consistency reliability within this study, so this information was reported as internal consistency, in keeping with COSMIN guidelines |
| **Construct: Fear of Falling** | | | | | | | |
| Fear of Falling Questionnaire-revised (FFQ-R) 15 item and 6 item  (Bower et al., 2015) | Adequate | EFA with principal axis factoring with oblique promax rotation | Kaiser-Meyer-Olkin = 0.82  RMSEA = 0.03  Four factors accounted for 10% or more of the common variance  In a post hoc analysis a two-factor solution was suggested, in which the authors removed reverse-scored items, weak correlations within the items and other latent factors resulting in a 6-item scale | Very good | **Cronbach’s alpha =** 0.76 for 15-item scale (hip fracture group)  **Cronbach’s alpha =** 0.80 for 6-item scale (hip fracture group) | Very good | **ICC** = 0.93 in hip fracture patients  Test-retest period: 3 days  Test-retest reliability for the six-item scale was ICC = 0.822 |
| FFQ-R  (Dautel et al., 2021) | Very good | CFA | Good model fit for the two-factor solution  X2/df = 3.335  RMSEA = 0.0000  Factor loading of at least 0.50 | Very good | **Cronbach’s alpha** = Scores:  Total = 0.79  Harm scale: 0.61  Threat scale: 0.78 | Adequate | Spearman correlations were 0.80, 0.93 and 0.53  Test-retest period: 3 weeks |
| Fear of Falling While Hospitalized Scale (Twibell et al., 2015) | Doubtful | Principal axis factor analysis | 77% of variance explained, eigenvalue = 5.4 (no mention of scree plot or of what was used to determine acceptable variance) | Very good | **Cronbach’s alpha** = .95 | Inadequate | Not reported – Cronbach’s alpha scores were reported as internal consistency reliability within this study, so this information was reported as internal consistency, in keeping with COSMIN guidelines |
| **Construct: Falls Risk Awareness** | | | | | | | |
| Self-Awareness of Falls in Elderly (SAFE) scale  (Birgill et al., 2022) | Very good | EFA & CFA – varimax rotation | Kaiser-Meyer-Olkin measure adequate at 0.771 and Bartlett test of sphericity was significant (*p* < 0.01).  Four factors accounted for 48.316% of the variance | Very good | **Cronbach’s alpha**  Scores:  Total = 0.811  Factor I = 0.859  Factor 2 = 0.618  Factor 3 = 0.577  Factor 4 = 0.508 | Adequate | **Test-retest reliability**  r = 0.575  (*p* <0.05) three weeks after first administration |
| SAFE scale  (Shyu et al., 2018) | Very good | EFA & CFA | The initial model was revised to four factors accounting for 61.15% of the variance | Very good | **Cronbach’s alpha**  Scores:  Total = 0.81  Factor I = 0.85  Factor 2 = 0.86  Factor 3 = 0.92  Factor 4 = 0.70 | Very good | **Interrater reliability**  Scores: Total = 0.83  Factor I = 0.84  Factor 2 = 0.86  Factor 3 = 0.92  Factor 4 = 0.70 |
| Self-Awareness of Falls Risk Measure (SAFRM)  (Mihaljic et al., 2014) | Adequate | PCA with oblique promax rotation – item factor loading was set at 0.4 and visual inspection of scree plot used | Kaiser-Meyer-Olkin measure adequate at 0.77 and Bartlett test of sphericity was significant (*p* < 0.01). Three factors accounted for 50.26% of the variance | Very good | **Cronbach’s alpha** scores: SAFRM Total = 0.92  Intellectual awareness = 0.89  Emergent awareness = 0.90  Anticipatory awareness = 0.86 | Very good | **ICC Clinicians**  SAFRM Total = 0.87 (*p* <0.001)  Intellectual awareness = 0.78  Emergent awareness = 0.61  Anticipatory awareness = 0.80  **ICC Patient & Clinicians**  SAFRM Total = 0.11 (*p* > 0.05)  Intellectual awareness = -0.02  Emergent awareness = -0.13  Anticipatory awareness = 0.39  (Low scores between patients & clinicians is expected as per study aims) |
| Falls Risk Awareness Questionnaire (FRAQ)  (Wiens et al., 2006) | Inadequate | Not reported | Not reported | Inadequate | Not reported | Inadequate | Not reported |
| Falls Risk Perception Questionnaire (FRPQ)  (Choi et al., 2020) | Adequate | EFA & varimax rotation | Kaiser-Meyer-Olkin measure adequate at 0.916 and Bartlett test of sphericity was significant (*p* < 0.01) at X2/df = 4092.418/351. This indicates that FRPQ is suitable for factor analysis. Three subfactors were extracted explaining 57.57% of the total variance | Very good | **Cronbach’s alpha** scores:  Total = 0.948  Factor I = 0.915  Factor 2 = 0.828  Factor 3 = 0.917 | Inadequate | Not reported – Cronbach’s alpha scores were reported as reliability within this study, so this information was reported as internal consistency, in keeping with COSMIN guidelines |
| **Construct: Outcome Expectancy** | | | | | | | |
| Consequences of Falling While Hospitalized Scale (Twibell et al., 2015) | Inadequate | Principal axis factor analysis | 29% of variance explained, eigenvalue = 3.5, authors decided to treat scale as a single-factor scale.  (no mention of scree plot or of what was used to determine acceptable variance) | Very good | **Cronbach’s alpha** = 0.84 | Inadequate | Not reported – Cronbach’s alpha scores were reported as internal consistency reliability within this study, so this information was reported as internal consistency, in keeping with COSMIN guidelines |
| Intention to Engage in Fall Prevention Scale (Twibell et al., 2015) | Doubtful | Principal axis factor analysis | 59% of variance explained, eigenvalue = 5.3 (no mention of scree plot or of what was used to determine acceptable variance) | Very good | **Cronbach’s alpha** = 0.90 | Inadequate | Not reported – Cronbach’s alpha scores were reported as internal consistency reliability within this study, so this information was reported as internal consistency, in keeping with COSMIN guidelines |

**Abbreviations**

ABC: Activities-Specific Balance Confidence Scale; ADLs: Activities of Daily Living; AUC: Area under the curve; BADL: Bristol Activities of Daily Living; CFA: Confirmatory Factor Analysis; CFI: Comparative Fit Index; CTT: Classical Test Theory; DASS-21: Depression, Anxiety and Stress Scale – 21 items; EFA: Exploratory Factor Analysis; FES: Falls Efficacy Scale; FES-I: Falls Efficacy Scale – International; FFQ-R: Fear of Falling Questionnaire-revised; FoF: Fear of Falling; FRAQ: Falls Risk Awareness Questionnaire; FRPQ: Falls Risk Perception Questionnaire (FRPQ); GDS: Geriatric Depression Scale; GFFM: Geriatric Fear of Falling Measure; ICC: Intraclass Correlation Coefficient; MFS: Morse Falls Scale; MMSE: Mini-Mental State Examination; PA: Percentage Agreement; PCA: Principal Component Analysis; PD: Parkinson’s Disease; POMA: Performance Oriented Mobility Assessment; RMSEA: Root Mean Square Error of Approximation; RSM: Rating Scale Model; SAFE: Self-Awareness of Falls in Elderly (SAFE) Scale; SAFRM: Self-Awareness of Falls Risk Measure; SCI: Spinal Cord Injury; SCI-FCS: Spinal Cord Injury-Falls Concern Scale;
