# Supplementary material for: Falls risk perception measures in hospital: a COSMIN systematic review
Source: J Patient Rep Outcomes. 2023 Jun 26;7:58. doi: 10.1186/s41687-023-00603-w (PMC10293508; doi:10.1186/s41687-023-00603-w)
Supplement: Supplementary file 5 — Additional file 5. Further measurement properties of included studies. [file 41687_2023_603_MOESM5_ESM.docx]

| **Scale & Author**  **Additional file 5: Further measurement properties of included studies** | **Cross-Cultural Validity**  **COSMIN**  **Rating Comments** | | **Measurement Error** | | **Hypothesis Testing for Construct Validity**  **COSMIN Rating Comments** | |
| --- | --- | --- | --- | --- | --- | --- |
| **Construct: Balance Confidence** | | | | | | |
| Activities-Specific Balance Confidence Scale (ABC) (Franchignoni et al., 2014) | N/A | N/A | ? | Not reported for long or short versions | Very good | ABC showed a high correlation with the Fear of Falling Measure (r = 0.85) and excellent correlation with its short versions (r > 0.93). |
| ABC-6P | N/A | N/A |  |  |  |  |
| ABC-6ON | N/A | N/A |  |  |  |  |
| ABC-5L | N/A | N/A |  |  |  |  |
| **Construct: Fall-Related Self-Efficacy** | | | | | | |
| Adapted version of the Falls Efficacy Scale (FES)  (Bula et al., 2008) | Doubtful | Insufficient information about translation to back-translation process. Low sample size according to COSMIN criteria | ? | Not reported | Very good | Pearson co-efficient assessed correlations between FES and other measures such as MMSE, POMA, previous falls, GDS and BADL scores |
| Modified-Falls Efficacy Scale (M-FES) (Perrot et al., 2018) | Very good | MFES-Fr has similar internal consistency to the original English version 0.95 (Hill, 1996) | ? | Not reported | Adequate | MANCOVA used to highlight significant differences between geriatric patients and community-dwelling adults, in which geriatric patients demonstrated lower self-efficacy |
| Falls Efficacy Scale (FES) (Hauer et al., 2010) | N/A | N/A | ? | Not reported | Doubtful | Comparison between groups according to age, gender, history of falls, fear of falling, TUG, vertigo and medication (Mann-Whitney U test, Kruskal-Wallis test) |
| Falls Efficacy Scale – International (FES-I) (Caronni et al., 2021) | N/A | N/A | ? | Not reported | Very good | Comparisons between  Mini-BESTest scale, TUG and FIM. Correlation between balance and concern about falling is weak in patients with poor balance |
| Falls Efficacy Scale – International (FES-I) (Hauer et al., 2010) | N/A | N/A | ? | Not reported | Doubtful | Comparison between groups according to age, gender, history of falls, fear of falling, TUG, vertigo and medication (Mann-Whitney U test, Kruskal-Wallis test) |
| Falls Efficacy Scale – International (FES-I) (Visschedijk et al., 2015) | N/A | N/A | Very good | SEM = 6.4  SDC = 17.7 | Doubtful  (time interval  unknown) | Several hypotheses stated with hypothesis confirmation with single-item FoF, HADS/A, GDS8 and fall history |
| Perform-FES  (Ferrer Soler et al., 2021) | N/A | N/A | ? | Not reported | Very good | Good correlations between the Perform-FES and other fear of falling scales (FES-I, Short FES-I, ABC, GFFM) with *P* <0.027  Criterion validity established with AUC 0.81 indicating that the Perform-FES outperformed other fear of falling scales *(P* < 0.05) |
| Spinal Cord Injury-Falls Concern Scale (SCI-FCS)  (Galante-Maia et al., 2021) | Very good | Very good sample size, similar measurement properties. | ? | Not reported | Inadequate | Other measures such as SCIM III but no comparison made between these measures and SCI-FCS-Brazil |
| Spinal Cord Injury-Falls Concern Scale (SCI-FCS)  (Pramodhyakul & Pramodhyakul, 2019) | Inadequate | Low sample size according to COSMIN criteria | ? | Not reported | Inadequate | Not reported |
| Spinal Cord Injury-Falls Concern Scale (SCI-FCS)  (Roaldsen et al., 2016) | Inadequate | Low sample size according to COSMIN criteria | Adequate | SEM = 2.6  SDC = 7.1  LoA = 6.1 to -8.2 | Inadequate | Not reported |
| Confidence to Perform Without Falling Scale (Twibell et al., 2015) | N/A | N/A | ? | Not reported | Doubtful | Significantly correlated with scores on similar scales (r=0.40-0.73; p=.001). |
| **Construct: Fear of Falling** | | | | | | |
| Fear of Falling Questionnaire-revised (FFQ-R) 15 item and 6 item  (Bower et al., 2015) | N/A | N/A | ? | Not reported | Adequate | Convergent validity: Pearson correlations with the Short FES-I and other measures were similar for both the 6-item and 15-item scale (r = 0.43 and 0.42 *p* < 0.001) |
| FFQ-R  (Dautel et al., 2021) | Adequate | Mostly comparable measurement properties to the English version. Different test-retest time periods | ? | Not reported | Adequate | Moderately correlated with the Short FES-I  (r = 0.51), as well as other measures |
| Fear of Falling While Hospitalized Scale (Twibell et al., 2015) | N/A | N/A | ? | Not reported | Doubtful | Significantly correlated with scores on similar scales (r=0.40-0.73; p=.001). |
| **Construct: Falls Risk Awareness** | | | | | | |
| Self-Awareness of Falls in Elderly (SAFE) scale  (Birgili et al., 2022) | Very good | Similar measurement properties to the English version (Shyu et al., 2018) | ? | Not reported | Adequate | Total scale correlation values of the items are between 0.294 and 0.601  (*p* <0.05) |
| SAFE scale  (Shyu et al., 2018) | N/A | N/A | ? | Not reported | Adequate | **Concurrent validity:** Negative correlation was observed with  r=-0.71 (*p*<.001), which means that people who experienced more falls in the previous year had lower self-awareness of falls risk |
| Self-Awareness of Falls Risk Measure (SAFRM)  (Mihaljic et al., 2014) | N/A | N/A | ? | Not reported | Very good | **Convergent validity:** Medium Spearman correlations with the SRSI awareness index (0.47) & the SRSI strategy generation index (0.47) (*p* < 0.001) |
| Falls Risk Awareness Questionnaire (FRAQ)  (Wiens et al., 2006) | N/A | N/A | ? | Not reported | Inadequate | Comparison made to a group of health professionals who completed the same survey to establish preliminary construct validity |
| Falls Risk Perception Questionnaire (FRPQ)  (Choi et al., 2020) | N/A | N/A | ? | Not reported | Very good | **Convergent validity:** Pearson correlation coefficient shows a positive correlation with the Korean FES-I (r = 0.735) and MFS (r = 0.392) |
| **Construct: Outcome Expectancy** | | | | | | |
| Consequences of Falling While Hospitalized Scale (Twibell et al., 2015) | N/A | N/A | ? | Not reported | Doubtful | Significantly correlated with scores on similar scales (r=0.40-0.73; p=.001). |
| Intention to Engage in Fall Prevention Scale (Twibell et al., 2015) | N/A | N/A | ? | Not reported | Inadequate | Not reported |

**Abbreviations:**

ABC: Activities-Specific Balance Confidence Scale; ADLs: Activities of Daily Living; FES: Falls Efficacy Scale; FES-I: Falls Efficacy Scale – International; FFQ-R: Fear of Falling Questionnaire-revised; FoF: Fear of Falling; FRAQ: Falls Risk Awareness Questionnaire; FRPQ: Falls Risk Perception Questionnaire (FRPQ); LoA: Limits of agreement; MIC: Minimal important change; PD: Parkinson’s Disease; SAFE: Self-Awareness of Falls in Elderly (SAFE) Scale; SAFRM: Self-Awareness of Falls Risk Measure; SCI: Spinal Cord Injury; SCI-FCS: Spinal Cord Injury-Falls Concern Scale; SDC: Smallest detectable change; SEM; SRSI: Self-regulation skills interview; Standard error of measurement; TUG: Timed up and Go test
